# Prevalence and genetic diversity of enteric viruses in Sub-Saharan Africa: a systematic review and meta-analysis

**DOI:** 10.1186/s12879-026-13391-7

**Published:** 2026-04-27

**Authors:** Ange Oho Roseline Badjo, Nongodo Firmin Kabore, Arsène Zongo, Justice Agbadu, Essia Belarbi, Abdoul-Salam Ouedraogo

**Affiliations:** 1 Laboratory of Emerging and Re-emerging Pathogens, Nazi Boni University, Bobo Dioulasso, 01 BP 1091 Burkina Faso; 2 Centre MURAZ, Institut National de Santé Publique, Bobo-Dioulasso, Burkina Faso; 3 Centre for International Health Protection, Public Health Laboratory Support, Robert Koch Institute, Unit 4, Berlin, Germany; 4 Charité – Universitätsmedizin Berlin, Berlin, Germany; 5 Centre for International Health Protection, Robert Koch Institute, Berlin, Germany

**Keywords:** Rotavirus, Norovirus, Astrovirus, Sapovirus, Gastroenteritis, Sub-Saharan Africa, Prevalence, Molecular epidemiology

## Abstract

**Background:**

Rotavirus A (RVA), norovirus (NoV), human astrovirus (HAstV), and sapovirus (SaV) are the main viruses responsible for acute gastroenteritis worldwide. Among them, RVA is generally the most prevalent, predominantly in Sub-Saharan Africa. With the introduction of RVA vaccines, several epidemiological changes have been reported in cases of viral gastroenteritis, particularly in Sub-Saharan Africa. Therefore, it is essential to understand the current burden and diversity of these viruses in order to guide public health interventions and vaccination strategies in the region.

**Objective:**

Our objective was to examine changes in prevalence data and circulating genotypes of RVA, NoV, SaV, and HAstV associated with acute gastrointestinal infections in both adults and children in Sub-Saharan Africa, based on studies published between 2010 and 2023.

**Methods:**

A systematic search was conducted in PubMed and Google Scholar to identify relevant studies published between 2010 and 2023, focusing exclusively on Sub-Saharan Africa. No restrictions were applied in terms of language or age group. Study selection, data extraction, and methodological quality assessment were performed using standardized procedures. Heterogeneity between studies was assessed using Cochrane’s Q test and the I² statistic in a random-effects model. Combined prevalence estimates for RVA, NoV, SaV, and HAstV were calculated using Comprehensive Meta-Analysis software.

**Results:**

A total of 55 studies from 19 countries in Sub-Saharan Africa were included. The combined prevalence was 31% for RVA, 14% for NoV, 12% for SaV, and 5% for HAstV. Overall, enteric viruses accounted for a combined prevalence of 22%. High heterogeneity (I² > 73%) was observed for most viruses. Genotyping data from 27 studies conducted in 13 countries showed high genetic diversity. RVA had 33 G/P combinations, the most common being G1P[8] and for NoV the GII.4 and GII.6 genotypes predominated. The high prevalence and genetic diversity of enteric viruses observed in this study underscore the continuing burden of viral gastroenteritis on public health in sub-Saharan Africa and confirm the need for ongoing surveillance to inform vaccination and control strategies.

**Supplementary Information:**

The online version contains supplementary material available at 10.1186/s12879-026-13391-7.

## Background

Diarrheal diseases remain a major public health issue worldwide, particularly in low- and middle-income countries (LMICs). In several settings, limited access to safe drinking water, sanitation, and healthcare contributes substantially to the disease burden, which may be further amplified in humanitarian or climate-affected contexts. Children under the age of five in LMICs, especially in Sub-Saharan Africa and South-East Asia, experience high morbidity and mortality rates associated with diarrheal diseases [1].

Acute gastroenteritis results from infections caused by a wide range of bacterial, viral and parasitic pathogens. Among viral agents, rotavirus A (RVA), norovirus (NoV), sapovirus (SaV) and Human astrovirus (HAstV) are recognized as the leading etiological agents of viral gastroenteritis globally [2]. Transmission occurs primarily through the fecal–oral route, facilitating widespread circulation in settings with suboptimal hygiene and sanitation conditions.

In Sub-Saharan Africa, RVA has historically been the most prevalent cause of severe viral gastroenteritis in young children, contributing to approximately one quarter to one third of acute gastroenteritis cases in the pre-vaccine era [1]. Following the introduction of RVA vaccines across many countries in the region, a substantial decline in RVA-associated hospitalizations has been documented [3]. However, accumulating evidence suggests epidemiological shifts, including changes in genotype distribution and an increasing relative of other enteric viruses.

Previous systematic reviews and meta-analyses conducted across Sub-Saharan Africa have documented a high burden of enteric viruses associated with acute gastroenteritis. For example, a meta-analysis estimated NoV prevalence at approximately 14% in young children, with GII (particularly GII.4) predominating in African settings before and after widespread rotavirus vaccination [4]. Similarly, regional syntheses have confirmed RVA and NoV as the leading viral pathogens, while SaV and HAstV are generally detected at lower but consistent frequencies, often ranging between 3% and 10%, depending on study population and diagnostic methodology [2, 5]. These studies highlight substantial heterogeneity across countries and sub-regions.

Genotype distribution analyses further reveal considerable viral diversity in Sub-Saharan Africa. For RVA, commonly G2P[4], G12[8], G1[8], G9[8] [6]. In contrast to the predominance of NoV GII.4 strains, multiple additional NoV genotypes and recombinant variants have also been documented across the region [4]. SaV circulation involves several genogroups, mainly GI and GII [7]. Regarding HAstV, the classic type 1 HAstV is the most common, with occasional detection of new variants such as MLB and VA [8]. This dynamic genetic landscape reflects ongoing evolution and poses challenges for surveillance and vaccine development.

Despite increasing numbers of country-specific studies and pathogens-focused reviews, data on enteric viral gastroenteritis in Sub-Saharan Africa remain fragmented. Most investigations target single pathogens, focus primarily on pediatric populations. Consequently, a comprehensive synthesis simultaneously addressing prevalence and genetic diversity of major enteric viruses across age groups in the post-RVA vaccine is lacking. In addition, methodological heterogeneity in study design, laboratory diagnostics, and genotyping approaches complicates direct comparisons across settings and over time.

To address these gaps, this systematic review and meta-analysis aim to synthesize available evidence on the prevalence and genetic diversity of major enteric viruses, including RVA, NoV, HAstV, and SaV, among both children and adults in Sub-Saharan Africa, based on studies published between 2010 and 2023. By providing an updated and regionally comprehensive overview, this study seeks to inform surveillance priorities and support the optimization of vaccination and public health control strategies for viral gastroenteritis in the region.

## Methods

### Study design and protocol registration

We performed a systematic review and meta-analysis using the PRISMA (Preferred Reporting Items for Systematic Reviews and Meta-Analysis) guidelines.

The protocol of this review was not registered in PROSPERO or any other public database, as the study was initiated before protocol registration was considered; retrospective registration was not pursued once study selection and data extraction had been completed.

### Study eligibility criteria

Eligible studies met the following criteria: (1) published between January 1, 2010 and December 31, 2023. (This period was selected to reflect the post-rotavirus vaccine era and the widespread adoption of molecular diagnostic techniques, which have significantly improved the detection and characterization of enteric viruses); (2) conducted in sub-Saharan African countries (Angola, Benin, Botswana, Burkina Faso, Burundi, Cameroon, Cape Verde, Central African Republic, Chad, Comoros, Democratic Republic of the Congo, Côte d’Ivoire, Djibouti, Equatorial Guinea, Eritrea, Eswatini, Ethiopia, Gabon, Gambia, Ghana, Guinea, Guinea-Bissau, Kenya, Lesotho, Liberia, Madagascar, Malawi, Mali, Mauritius, Mauritania, Mozambique, Namibia, Niger, Nigeria, Uganda, Republic of the Congo, Rwanda, Sao Tome and Principe, Senegal, Seychelles, Sierra Leone, Somalia, Soudan, South Sudan, South Africa, Tanzania, Togo, Zambia, Zimbabwe); focused on at least one of the four viruses of interest (RVA, NoV, SaV, HAstV); (3) investigated symptomatic patients (regardless of age or sex); (4) reported laboratory-confirmed infections (based on positive serological results and/or molecular test results). Only articles with full text available have been included.

The exclusion criteria were: literature reviews, meta-analysis, environmental studies, animal studies, articles with duplicate or overlapping data, articles without full text available or studies not directly related to the target viruses.

### Search strategy

The bibliographic search was conducted between March and May 2025 using PubMed and Google Scholar, selected for their comprehensive coverage of biomedical and public health literature. The search was restricted to studies published up to December 31, 2023, in order to include the most recent complete year of data and ensure methodological consistency and reproducibility.

In PubMed, the following search terms were used:

(Norovirus OR Rotavirus OR Astrovirus OR Sapovirus) AND (Diarrhea OR Diarrhea OR Gastroenteritis) AND (Humans[Mesh] OR human OR patient) AND (Sub-Saharan Africa OR Africa South of the Sahara OR Nigeria OR Kenya OR South Africa OR Ghana OR Ethiopia OR Uganda OR Tanzania OR Senegal OR Burkina Faso OR Mali OR Cameroon OR “Ivory Coast” OR “Côte d’Ivoire” OR “Democratic Republic of Congo” OR Zambia OR Zimbabwe OR Madagascar OR Chad OR Sudan OR “South Sudan” OR Malawi OR Togo OR Benin OR Rwanda OR Burundi OR Mozambique OR Botswana OR Namibia OR Gabon OR “Sierra Leone” OR Liberia OR Congo OR Gambia OR Niger OR Mauritania OR Guinea OR “Guinea-Bissau” OR Eswatini OR Lesotho OR Comoros OR Seychelles OR Djibouti OR Eritrea) AND (Prevalence OR Epidemiology OR Genotyping OR “Genetic diversity” OR “Molecular characterization”) NOT (Review[Publication Type] OR “Systematic Review” OR “Meta-Analysis”) NOT (Animals[Mesh] OR animal OR veterinary) AND (“2010/01/01“[Date - Publication]: “2023/12/31“[Date - Publication]).

In Google Scholar, the following search terms were used:

(“Norovirus” OR “Rotavirus” OR “Astrovirus” OR “Sapovirus”) AND (“Diarrhea” OR “Diarrhea” OR “Gastroenteritis”) AND (“Sub-Saharan Africa” OR “Africa” OR “Burkina Faso” OR “Nigeria” OR “Kenya” OR “Ghana” OR “Ethiopia” OR “South Africa” OR “Gabon”) AND (“Prevalence” OR “Epidemiology” OR “Genotyping” OR “Genetic diversity” OR “Molecular characterization”) -veterinary -animal -review -“systematic review” -“meta-analysis” -“Environmental surveillance” -“Environmental study” -“wastewater surveillance” -“waterborne transmission”.

Manual searches: In addition to the initial automated search, 20 additional articles were identified through targeted searches on Google Scholar, focusing on specific countries or virus names (NoV, RVA, SaV and HAstV) to ensure comprehensive coverage.

### Selection process, data extraction and quality assessment

Two independent reviewers screened the titles and abstracts for eligibility. The Full texts of potentially relevant studies were then independently assessed by these same reviewers. Discrepancies in study inclusion were resolved through discussion, and if consensus was not reached, a third reviewer made the final decision. After the title and abstract were screened, the full text was screened.

For each eligible study, data were extracted using a customized Microsoft Excel spreadsheet. The following variables were collected when available: country and study period, population characteristics, viruses detected, detection methods, prevalence rates, and genotypic diversity. Case-control studies were excluded from the analysis of pooled prevalence because their design does not allow for a representative estimate of the frequency of the viruses studied. However, genetic data from control groups were taken into account when assessing the genotypic diversity of the viruses identified.

The methodological quality of the included studies was assessed using the Joanna Briggs Institute (JBI) critical appraisal checklists, tailored to each study design [9]. For cross-sectional studies and prevalence studies, the checklists included eight or nine items covering clear inclusion criteria, appropriate sampling, representativeness of the target population, validity and reliability of outcome measurement, and appropriate statistical analysis.

Cohort studies were assessed using an 11-item checklist, focusing on baseline comparability, validity and reliability of exposure measurement, identification and management of confounding factors, completeness of follow-up, and appropriateness of analysis.

Each item was assigned a score of 1 if the checkpoint was met (“Yes”) and 0 if it was not (“No” or “Unclear”). According to the appreciate JBI checklist (ranging from 8 to 11 items depending of the study design), studies were categorized as high quality (≥ 80% of the maximum score), moderate (60–79%) or low quality (< 60%).

The study quality assessment helped identify potential sources of bias and informed the interpretation of the pooled results. Details of the risk of bias assessment are provided in Supplementary Tables 1–3.

### Data analysis

We performed statistical analyses using R (version 4.4.1) and STATA and Stata/MP 15.1 (StataCorp, Texas, USA). The meta and metafor packages were applied to extract the effect sizes and their corresponding standard errors for each study included in this review.

To account for variability across studies, we used a random-effects meta-analysis model to generate pooled effect sizes and their 95% confidence intervals (CI). Where appropriate, subgroup analyses were also carried out to examine potential sources of heterogeneity, such as geographic region, virus type, and detection methods. Forest plots were generated, and statistical significance was assessed using appropriate tests (Z-test for pooled effects; Cochran’s Q test for heterogeneity); a p-value less than 0.05 was considered statistically significant.

Statistical heterogeneity across studies was assessed using Cochran’s Q test and quantified by the I² statistic to estimate the proportion of total variation attributable to between-study heterogeneity rather than chance. To explore potential sources of heterogeneity, a meta-regression was conducted to examine whether the detection methods influenced the pooled prevalence estimates. Reverse transcription polymerase chain reaction (RT-PCR) was used as the reference category, as it is considered the gold standard for the detection of enteric viruses and was the most frequently reported method in the included studies. Dummy variables were created for EIA (Enzyme ImmunoAssay), ELISA (Enzyme-Linked Immunosorbent Assay), (ICG Immunochromatographic Test), and EIA combined with RT-PCR.

Publication bias was assessed using Begg’s adjusted rank correlation test and Egger’s regression asymmetry test applied to the pooled effect sizes. A funnel plot was also generated to visually inspect the symmetry of the included studies. A p-value below 0.05 was considered indicative of potential small-study effects or publication bias.

## Results

**Fig. 1:**
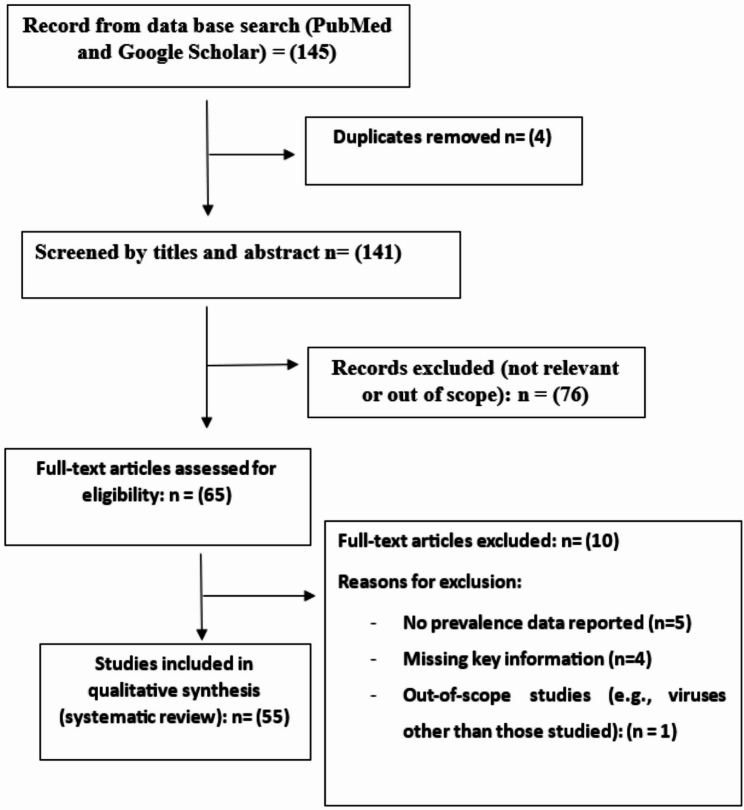
PRISMA flow chart of study selection and criteria. A total of 145 articles were identified through PubMed and Google Scholar. After removing 4 duplicates, 141 records were screened, and 76 were excluded as not relevant or out of scope. 65 articles were assessed in full text, and 55 were includes in final synthesis after excluding 10 studies for reasons such as lack of prevalence data, missing key information, or being out of scope

In this systematic review, a total of 145 articles were identified through the initial search in PubMed and Google Scholar. After removing duplicates, 141 (97%) were retained for screening. Based on the examination of the titles and abstracts, 65 articles (43%) were selected for full-text assessment. After detailed review, 10 studies were excluded due to reasons detailed in Fig. 1.

A total of 55 studies met the eligibility criteria and were included for qualitative synthesis. Of these, 5 case-control studies were excluded from the pooled prevalence meta-analysis but were considered in the assessment of genetic diversity. Thus, 50 studies were included in the quantitative synthesis.

### Characteristics of included studies

Table 1 provides a summary of studies included in the review, categorized by Sub-Saharan African regions and countries. West Africa accounted for the majority of the studies (56%), with Burkina Faso and Nigeria contributing for 11 and 10 publications respectively. Southern Africa accounted for 22% of the studies; among East African countries. East and Central Africa were less represented, accounting for 18.1% and 4% of the included studies, respectively.

**Table 1:** Summary of included studies according to geographic region and country in Sub-Saharan Africa

Region	Country (No. of studies per country)	No. of studies	% by Region
West Africa	Burkina Faso (11), Nigeria (10), Ghana (3), Benin (1), Côte d’Ivoire (1), Niger (1), Senegal (1)	28	56%
East Africa	Kenya (5), Tanzania (1), Ethiopia (2), Zambia (1)	9	18%
Central Africa	Gabon (1), Cameroon (1)	2	4%
Southern Africa	South Africa (6), Mozambique (2), Eswatini (1), Botswana (1), Zimbabwe (1)	11	22%
TOTAL		50	100%

### Etiology of viral gastroenteritis

We included 50 studies from Sub-Saharan Africa, covering four sub-regions: West, East, Central, and Southern Africa. Most studies focused on children under 5 years of age (46/50; 92%), though a few included older children or all age groups. Of the 50 studies, RVA was the most investigated virus, reported in 41 studies (62%). NoV was reported in 30% of the studies, while HAstV and SaV were less reported in 4 (8%) and 6 (12%) studies, respectively. The most frequently used detection methods in the included studies included were RT-PCR (34%) and EIA (30%), followed by ELISA (24%) and immunochromatographic tests (ICG) (10%). One study (2%) combined EIA and RT-PCR.

#### West Africa

West Africa was the most represented region, with studies conducted in countries such as Burkina Faso, Ghana, Nigeria, Côte d’Ivoire, Niger, Benin, and Senegal [10–37]. Reported RVA prevalence ranged from 14.6% to 70%, while NoV prevalence ranged from 6% to 40.9%, SaV from 10.3% to 18%, and HAstV from 4.9% to 9%.

#### East Africa

In East Africa, studies were conducted in Ethiopia, Kenya, Tanzania, and Zambia [38–45]. RVA prevalence ranged from 10.5% to 38.4%, NoV from 10.9% to 13.2%, while SaV and HAstV were only reported in few studies (see Table 2).

#### Central Africa

Central African studies, mainly from Cameroon and Gabon, reported RVA prevalence ranging from 27.1% to 41%, while NoV and SaV were documented by only one [46, 47] (see Table 2).

#### Southern Africa

In Southern Africa, studies were conducted in South Africa, Zimbabwe, Mozambique, Eswatini, and Botswana [48–59]. RVA prevalence varied widely from 3.8% to 60.7%, NoV ranged from 2.7% to 16%, while HAstV was documented in only two countries and SaV was rarely reported (Table 2).

The Table 2 presents the reported prevalence rates for RVA, NoV, SaV and HAstV in the different Sub-Saharan African regions.

**Table 2:** RVA, NoV, SaV and HAstV prevalence rates in Sub-Saharan Africa

Country	Year of publication	Population Age group	Method	Number of Participants	Viruses Detected	Prevalence(s)	Reference
**West Africa**							
Benin	2020	< 5 years	EIA	420	RVA	39.8%	[10]
Burkina Faso	2011	< 5 years	ELISA	309	RVA	32.4%	[11]
Burkina Faso	2017	≤ 5 years	ICG	332	RVA	64.2%	[22]
Burkina Faso	2011	< 5 years	ICG	447	RVA	33.8%	[31]
Burkina Faso	2016	< 5 years	RT-PCR	263	RVA	63.5%	[32]
					NoV GI	2.7%	
					NoV GII	18.2%	
					SaV	10.3%	
					HAstV	4.9%	
Burkina Faso	2012	< 5 years	ELISA	80	RVA	70%	[33]
Burkina Faso	2020	< 5 years	Mixed	146	RVA NoV	14% 20%	[34]
Burkina Faso	2018	< 5 years	EIA; RT-PCR	154	RVA NoV	44% 23%	[35]
Burkina Faso	2015	< 5 years	RT-PCR	309	NoV	18%	[36]
Burkina Faso	2014	< 5 years	ICG	103	RVA	17.5%	[37]
Burkina Faso	2019	< 5 years	RT-PCR	213	RVA	14.6%	[12]
Burkina Faso	2021	< 5 years	RT-PCR	293	NoV	21.2%	[13]
Côte d’Ivoire	2018	< 5 years	EIA	684	RVA	27.1%	[14]
Ghana	2011	< 5 years	ELISA	143	RVA	58%	[15]
Ghana	2023	< 5 years	RT-PCR	263	RVA	14.8%	[16]
Ghana	2013	0–59 months	EIA	2277	RVA	48.2%	[60]
Niger	2019	< 5 years	EIA	84	RVA	21.4%	[18]
Nigeria	2013	< 5 years	RT-PCR	71	RVA	22.5%	[25]
Nigeria	2019	1–59 months	EIA	100	RVA	30%	[19]
					NoV	11%	
Nigeria	2023	< 5 years	ICG	109	RVA	69.17%	[20]
					NoV GI	1.67%	
					NoV GII	8.33%	
Nigeria	2012	< 5 years	ELISA	161	HAstV	40.4%	[21]
Nigeria	2022	0–59 months	EIA	735	RVA	20.8%	[23]
Nigeria	2022	< 5 years	ELISA	275	RVA	25.6%	[24]
Nigeria	2022	1–59 months	ELISA	414	RVA	43%	[27]
Nigeria	2023	< 5 years	RT-PCR	100	NoV	6%	[28]
					HAstV	9%	
Nigeria	2019	< 5 years	RT-PCR	2694	RVA	46%	[29]
Nigeria	2017	< 5 years	ELISA	467	RVA	31%	[26]
Senegal	2016	0–59 months	ELISA	740	RVA	30.6%	[30]
**East Africa**							
Ethiopia	2022	< 5 years	RT-PCR	38	RVA	10.5%	[39]
					NoV	13.2%	
					SaV	7.9%	
					HAstV	5.3%	
Ethiopia	2018	< 5 years	ELISA	112	RVA	18%	[40]
Kenya	2015	< 5 years*	RT-PCR	260	RVA	31%	[61]
Kenya	2018	< 5 years	EIA	3779	RVA	27.3%	[38]
Kenya	2020	< 5 years	RT-PCR	817	RVA	13.8%	[43]
					NoV GII	10.9%	
					SaV	2.6%	
					HAstV	3.2%	
Kenya	2020	< 13 years	EIA	248	RVA	22.2%	[62]
Kenya	2017	< 5 years	EIA	298	RVA	31.5%	[42]
Tanzania	2023	0–59 months	RT-PCR	146	RVA	38.36%	[41]
Zambia	2018	< 5 years	ELISA	1466	RVA	24.7%	[45]
**Central Africa**							
Cameroon	2014	< 5 years	EIA	2444	RVA	41%	[47]
Gabon	2015	< 5 years	RT-PCR	317	RVA	27.1%	[46]
					NoV GI	9.1%	
					NoV GII	13.9%	
					SaV	9.5%	
					HAstV	6.3%	
**Southern Africa**							
Botswana	2020	< 5 years	ELISA	200	RVA	11%	[48]
Eswatini	2018	< 5 years	EIA	596	RVA	60.7%	[49]
Mozambique	2021	≤ 15 years	EIA	2126	RVA	23.4%	[52]
					NoV	2.7%	
					HAstV	3.3%	
Mozambique	2018	< 5 years	EIA	1296	RVA	26.3%	[53]
South Africa	2023	< 5 years	RT-PCR	275	NoV	15%	[54]
South Africa	2021	< 5 years	RT-PCR	166	HAstV	7.23%	[55]
South Africa	2020	All ages	RT-PCR	80	NoV	16%	[63]
South Africa	2018	All ages	RT-PCR	340	NoV	12.9%	[51]
South Africa	2016	< 5 years	ELISA	1792	RVA	21.7%	[58]
South Africa	2018	< 5 years	EIA	237	RVA	3.8%	[59]
					NoV	5.1%	
Zimbabwe	2014	< 5 years	EIA	3728	RVA	48.5%	[50]

Note: * HIV+, EIA=Enzyme ImmunoAssay, ELISA= Enzyme-Linked Immunosorbent Assay, ICG= Immuno Chromatographic Test, RVA: Rotavirus, NoV: Norovirus, HAstV: Astrovirus, SaV: Sapovirus

### Pooled prevalence estimates

**Fig. 2:**
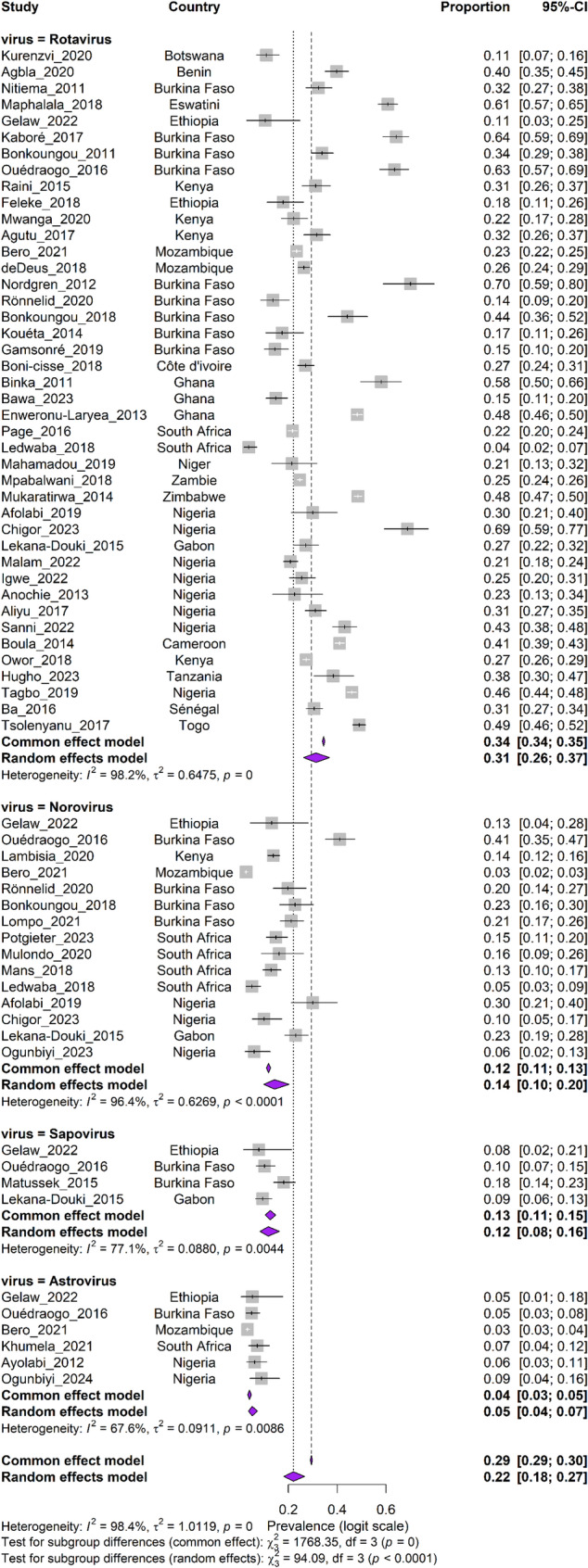
Forest plot showing pooled prevalence and heterogeneity measures of RVA, NoV, HAstV and SaV in Sub-Saharan Africa. Each line represents an individual study, with its prevalence (point) and 95% CI (horizontal bars). The purple diamonds indicate estimates pooled according to random effects model, presented separately for each virus (RVA, NoV, SaV and HAstV). Heterogeneity values (I2) and subgroup tests are shown at the end of the graph

Overall, the pooled prevalence estimates varied considerably between enteric viruses. RVA showed the highest pooled prevalence (31%, 95% CI: 26%–37%), followed by NoV (14%, 95% CI: 10%–20%), SaV (12%, 95% CI: 8%–16%) and HAstV (5%, 95% CI: 4%–7%). The combined pooled prevalence for all enteric viruses was 22% (95% CI: 18%–27%). Substantial heterogeneity was observed within each subgroup (I² ranging from 67% to 98%), and heterogeneity between subgroups was statistically significant (Q = 94.1, df = 3, *p* < 0.001). Detailed estimates are provided in Fig. 2.

#### Impact of vaccine introduction on rotavirus prevalence

**Fig. 3:**
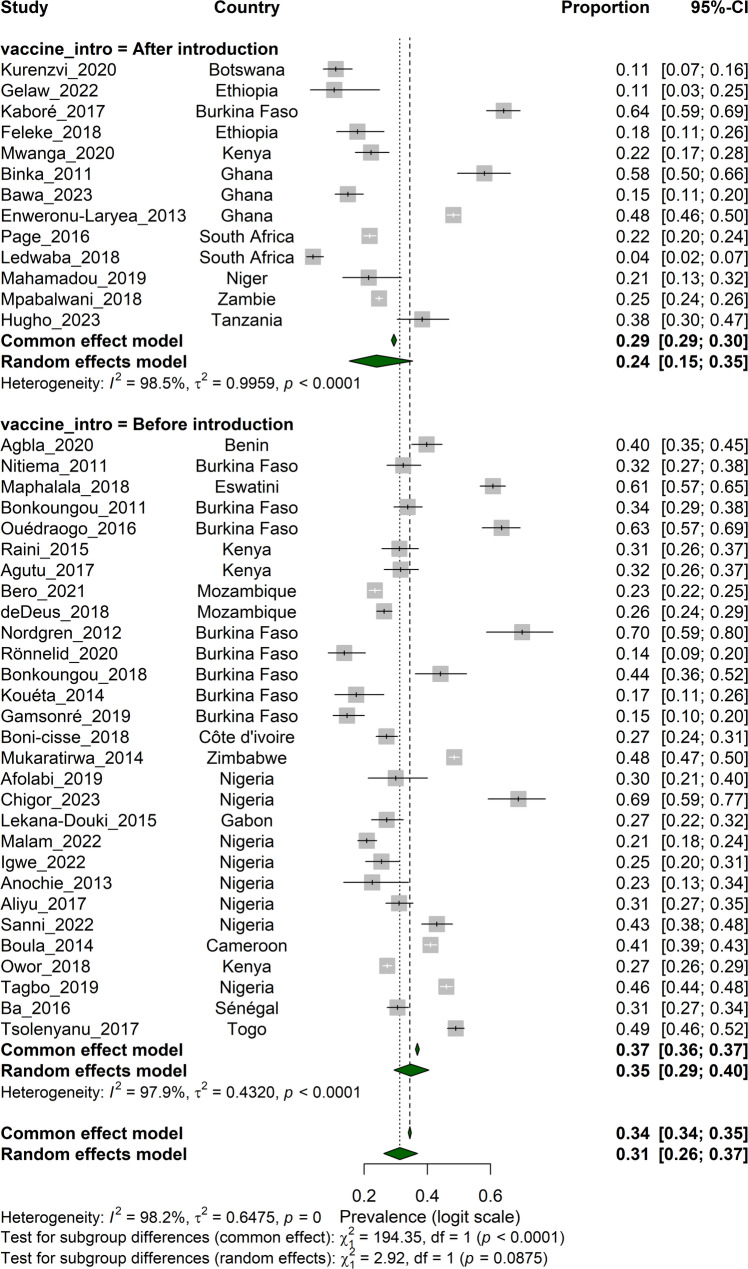
Forest plot for impact of RVA vaccine introduction on RVA prevalence. Each line corresponds to a study, with its observed prevalence (point) and 95% CI (horizontal bars). The green diamonds represent the combined estimates according to a random effects model, presented separately for the periods before and after the introduction of the RVA vaccine in the country

The meta-analysis assessed the pooled prevalence of RVA infection before and after the introduction of the vaccine in the countries included. The classification was based on each country’s official vaccine introduction date. The pooled prevalence before vaccine introduction was 35.0% [95% CI: 29%–40%], compared to 24% [95% CI: 15%–35%] after vaccine introduction (*p* < 0.001). Subgroup heterogeneity remained high in both periods (I² > 97%) (Fig. 3).

A meta-regression analysis evaluating the effect of vaccine introduction on RVA prevalence showed a reduction of approximately 8% points in the post-vaccine introduction, compared to the pre-vaccine period. However, this reduction was not statistically significant (Coef. =-0.079; 95% CI: [-0.191; 0.033]; *p* = 0.163). Considerable residual heterogeneity was still observed (I² = 98.5%), and the adjusted R² indicated that vaccine introduction explained only 2.5% of the between-study variance.

From all countries includes, only Burkina Faso had data covering pre- and post-vaccine periods, allowing for a country-specific meta-regression. In the case, the introduction of RVA vaccine did not show a significant effect on the effect size (β = 0.28; 95% CI: -0.26 to 0.82; *p* = 0.26).

#### Publication bias and qualitative risk of bias assessment

**Fig. 4:**
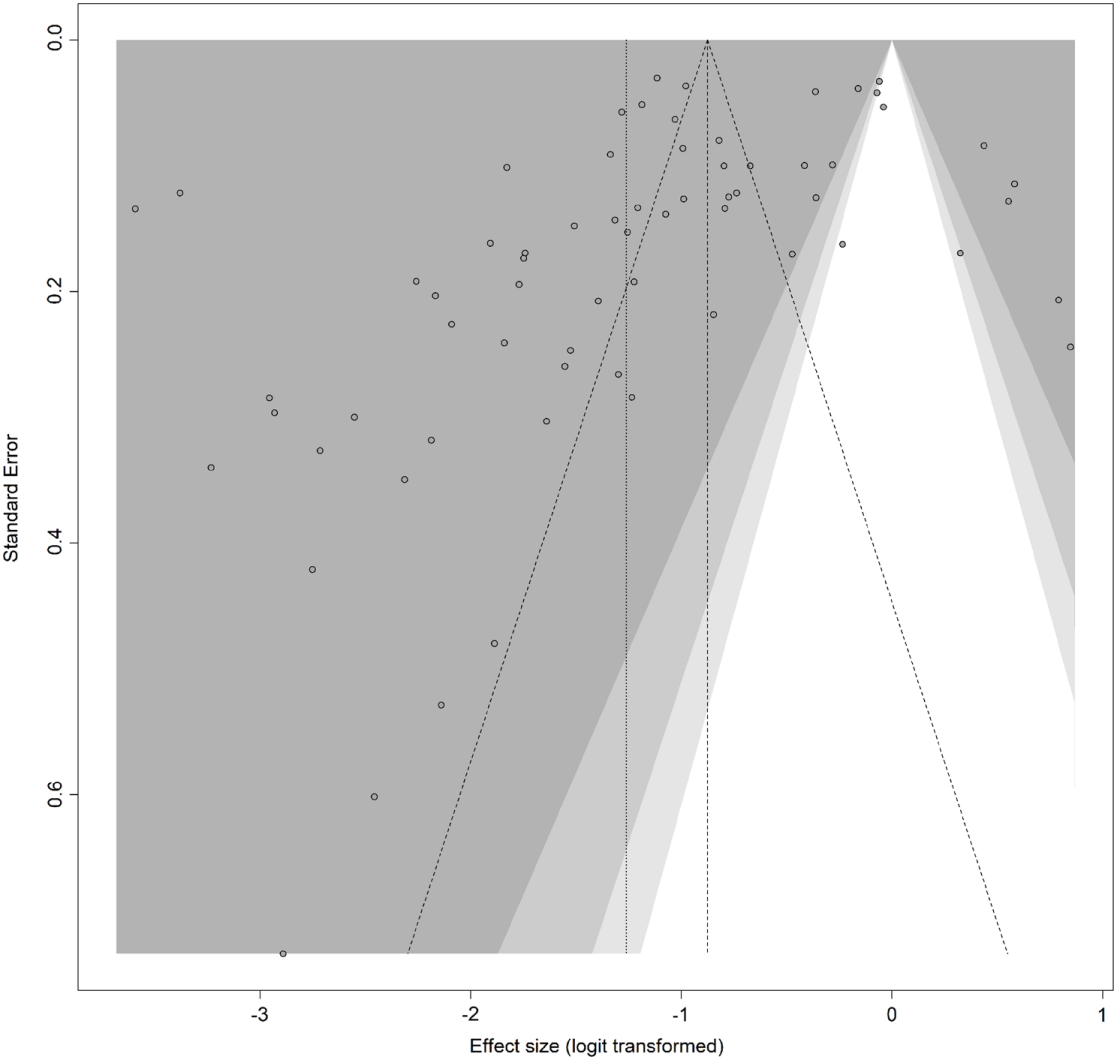
Funnel plot for assessment of publication bias among studies included in the meta-analysis. Each point represents an individual study, positioned according to its effect size (x-axis, Effect size, transformed into logit) and its standard error (y-axis). The vertical line corresponds to the estimated combined effect, while the slanted lines delimit the 95% CI. The gray areas illustrate the expected dispersion of studies in the absence of publication bias. The asymmetry observed in the graph suggests a possible publication bias or significant residual heterogeneity between studies

Egger’s regression asymmetry test indicated significant asymmetry of the funnel plot (intercept = 7.04; *p* = 0.002). In contrast, Begg’s rank correlation test did not show significant correlation between effect sizes and their standard errors (Kendall’s tau-b = 0.095; *p* = 0.264). Visual inspection of the funnel plot confirmed this asymmetry (Fig. 4). As 66 effect sizes were extracted from 50 articles due to the inclusion of multiple viruses per study, this result should be interpreted with caution, since multiple estimates per study may artificially increase the detection of asymmetry.

Of the 50 studies included, 68% (*n* = 34) were judged to have a low risk of bias, 26% (*n* = 13) a moderate risk, and 8% (*n* = 3) a high risk of bias.

### Leave-one-out sensitivity analysis

A leave-one-out sensitivity analysis was performed to evaluate the influence of each individual study on the overall pooled prevalence estimate. The pooled prevalence ranged from 25% to 26% depending on which study was excluded. The corresponding 95% CI showed lower limits between 0 and 60%, and upper limits between 34 and 80%.

### Subgroup analyses

#### Subgroup analysis by detection method

To explore whether the detection method could explain the observed heterogeneity, a meta-regression was performed. The meta-regression indicates that, compared to RT-PCR, the use of EIA, EIA combined with RT-PCR, ELISA, or ICG did not show a statistically significant difference in prevalence estimates (all *p* > 0.1). The model failed to account for the high residual heterogeneity (adjusted R^2^ =-1.8%; residual I^2^= 99.3%).

#### Subgroup analysis by region

**Fig. 5:**
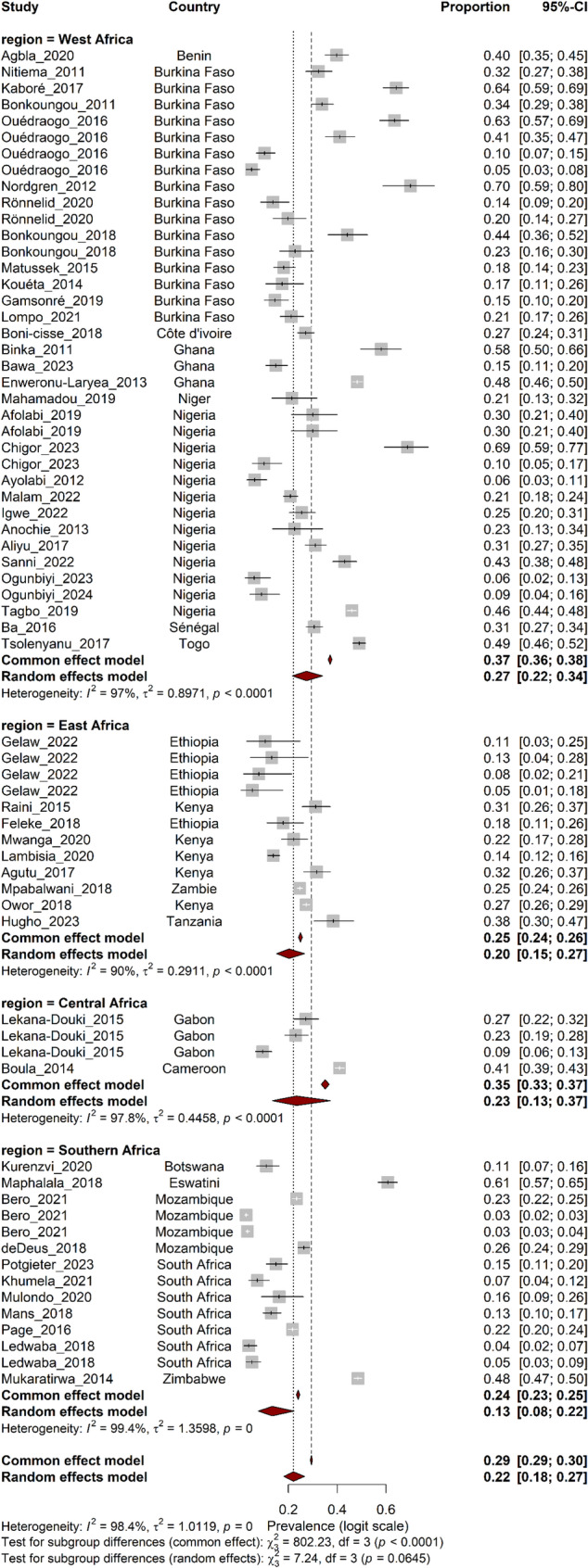
Forest plot showing pooled prevalence estimates stratified by region. Each line represents an individual study classified by region (West, East, Central, and Southern Africa), country, and author. The squares indicate the estimated prevalence proportion, with their size reflecting the weight of the study in the meta-analysis. The horizontal bars represent the 95% CI. The red diamonds indicate the overall estimates for each region according to the fixed-effect model (“Common effect model”) and random-effects model (“Random effects model”). Some studies appear several times because they report separate data on several viruses at once. The statistics at the bottom indicate the heterogeneity (I^2^) between studies in each region as well as an overall test of differences between regional subgroups

A subgroup meta-analysis was conducted to estimate the pooled prevalence by geographic region. The forest plot in Fig. 5 illustrates the pooled estimates and confidence intervals for each region. A total of 66 effect sizes were included, covering studies from West Africa, Central Africa, East Africa, and Southern Africa.

Overall, the pooled prevalence across all regions was 22% (95% CI:18%–27%). West Africa accounted for the largest proportion of studies (54.5%) with a pooled prevalence of 27% (95% CI: 22%–34%). The pooled prevalence was 23% (95% CI: 13%–37%) for Central Africa, 20% (95% CI: 15%–27%) for East Africa, and 13% (95% CI: 8%–22%) for Southern Africa.

There was considerable heterogeneity observed across studies (I² = 98.4%, τ² = 1.0119, *p* < 0.05). When assessing differences between subgroups by geographic region, the test under a random-effects model was not statistically significant (Q = 7.24, df = 3, *p* = 0.0645).

#### Cross-analysis by region and virus

We conducted a stratified meta-analysis combining region and detected virus to explore prevalence variability. The results show significant differences between subgroups (*Q-between*, *p* < 0.001). RVA prevalence was generally higher in West Africa (36.6%) and Central Africa (34.3%) compared to Southern Africa (27.9%) and East Africa (26.1%). For NoV, prevalence showed minor regional variation (13.6% in West Africa vs. 14% in Southern Africa). However, for HAstV and SaV, some estimates are based on very few studies, which limits precision (some subgroups include only a single study).

### Distribution of genotypes

In this systematic review, 55 studies were included in total, of which 27 provided genotyping data for at least one of the target enteric viruses. These data were obtained from 13 countries in Sub-Saharan Africa: Benin, Botswana, Burkina Faso, Côte d’Ivoire, Ethiopia, Gabon, Ghana, Kenya, Nigeria, South Africa, Tanzania, Togo, and Zimbabwe.

#### Distribution of RVA genotypes

RVA genotype data were found in eleven countries: Benin, Botswana, Burkina Faso, Ethiopia, Côte d’Ivoire, Ghana, Gabon, Nigeria, Togo, Tanzania, and Zimbabwe [10, 14–17, 25, 29, 31–35, 38, 39, 42, 46, 48, 50, 58, 62, 64, 65]. A total of thirty-three different G/P genotype combinations were identified in this study. G1P[8] was the most frequently notified genotype, detected in all eleven countries, followed by G2P[4] and G12P[8], each found in nine countries. Other frequently reported genotypes were G3P[6], G9P[8], and G2P[6], all observed in at least six countries. A wide range of less common and emerging genotypes were also identified, such as G8P [14], G12P [4], and mixed or non-typeable combinations (such as G1 + G2P [4,8], GNTP[6], GxPNT), particularly in Kenya and Burkina Faso (See Supplementary Table 4) for comprehensive genotype distribution tables).

#### Distribution of NoV genotypes

Among the studies included in this review, NoV genotyping data were available from three countries: Burkina Faso, Ethiopia, and South Africa [32, 34, 35, 39, 51, 63]. Genogroup II (GII) was the most frequently reported genotypes, with GII.4 and GII.6 identified in all three countries. Several other GII were also detected, including, GII.1, GII.2, GII.3, GII.9, GII.12, GII.14 and GII.16. Genogroup I (GI) genotypes were less commonly reported but included GI.1, GI.2, GI.3, GI.5, and mixed strains like GI.f/I.3. Burkina Faso had the highest diversity of NoV genotypes, followed by South Africa and Ethiopia (See Supplementary Table 5 for comprehensive genotype distribution tables).

#### Distribution of SaV genotypes

SaV genotypes were notified from three Sub-Saharan African countries: Burkina Faso, Ethiopia, and Gabon [32, 36, 39, 66]. A variety of genotypes were identified across these countries, reflecting moderate genetic diversity. GII.1 was the most frequently reported genotypes, detected in all three countries, as well as GII.5 and GII.6, found in Ethiopia, Gabon, and Burkina Faso. Several GI (GI.1, GI.2, GI.3, GI.4) were detected principally in Gabon and Burkina Faso, as well as other GII genotypes (GII.2, GII.3, GII.4). Notably, uncommon genotypes such as GIV.1 and GV.1 were exclusively detected in Burkina Faso (See Supplementary Table 6).

#### Distribution of HAstV genotypes

HAstV genotyping data were available from four countries in Sub-Saharan Africa: Burkina Faso, Ethiopia, Gabon and South Africa [32, 39, 46, 55, 66]. HAstV-1 was the most common genotype, detected in three countries (South Africa, Burkina Faso, and Gabon). Other classic genotypes such as HAstV-2, HAstV-4, HAstV-5, and HAstV-8 were also identified in at least one of the included countries. Gabon had the greatest genetic diversity, with both classic genotypes and emerging non-classic strains such as HAstV-VA2 and MLB1 (See Supplementary Table 7).

## Discussion

Diarrheal diseases have long represented one of the principal global causes of morbidity and mortality, with the greatest burden observed among children under five years of age. Viruses are often responsible for gastrointestinal infections, and RVA has long been the predominant virus throughout the world. Other enteric viruses, such as NoV, HAstV and SaV, have been well described in several industrialized countries, but little information is available on their epidemiology in LMICs, especially in Sub-Saharan Africa.

The present systematic review and meta-analysis included 55 studies reporting the prevalence of different enteric viruses (RVA, NoV, HAstV and SaV) associated with acute gastrointestinal infections in children and adults in Sub-Saharan African countries, published between 2010 and 2023. Only 18 countries provided data on at least one of the viruses studied. West Africa contributed the highest number of studies among Sub-Saharan African regions, with Burkina Faso and Nigeria reporting 10 and 11 studies, respectively.

The pooled prevalence of RVA in our study (2010–2023), which included all age groups, was 31%, which is slightly higher than that reported in a systematic review covering the period 2007–2019 in children under five years old (26%) [67], but lower than surveillance data collected between June 2006 and December 2008 among children under five (40%) [68]. These results suggest that, despite the introduction of the RVA vaccine, which primarily targets G1P[8], RVA continues to be a major cause of viral gastroenteritis in the region. However, it is difficult to directly assess the impact of vaccination without stratifying data into pre- and post-vaccine introduction periods.

NoV was the second most prevalent virus in this review, with a pooled prevalence of 14%.This confirms the predominance of NoV as a major cause of both sporadic cases and outbreaks of gastroenteritis in Sub-Saharan Africa [69, 70]. A review evaluating the impact of introducing RVA vaccines in Africa on the prevalence and genetic diversity of NoV reported pooled estimates of NoV 15% before and 13% after the introduction of RVA vaccination [4]. These results, which are similar with our study, indicate that NoV remains a major cause of viral gastroenteritis and highlight the need to strengthen surveillance and preventive measures against NoV alongside existing RVA vaccination programs. An additional study conducted in Botswana among hospitalized children under five years of age between 2013 and 2015, identified after completion of the screening process, reported a NoV prevalence of 9.3% [71]. Although not included in the quantitative synthesis, the estimate falls within the range observed in our meta-analysis.

The pooled estimates of SaV and HAstV prevalence were lower (12% and 5%, respectively), in our study, and remain under detected in routine surveillance due to limited diagnostic capacity in many African settings [68]. However, their contribution to the overall burden of pediatric diarrhea should not be underestimated, given their potential to cause outbreaks and co-infections, which adds to the complexity of the epidemiology of viral gastroenteritis [72–74].

The significant heterogeneity observed both within and between virus subgroups highlights important variation in study designs, population age groups, sampling strategies, seasonal patterns, and diagnostic approaches. Such variability points the need for standardized surveillance protocols and improved diagnostic capacity in Sub-Saharan Africa.

Despite the significant heterogeneity in our study, the combined pooled prevalence for all enteric viruses (22%) confirms their major contribution to the burden of diarrheal diseases in Sub-Saharan Africa over the past decade. These findings highlight the need for public health strategies, including strengthening of vaccination programs, the promotion of water, sanitation and hygiene (WASH) interventions, and expanding molecular surveillance systems.

A stratified analysis by virus and geographic region was also performed. This cross-analysis revealed significant differences in prevalence between regions. These regional variations may be due by a mix of factors, including differences in surveillance intensity, population health conditions, climate, and access to safe drinking water and sanitation across regions. it is also important to note that for HAstV and SaV, data are still scare, with very limited number of studies, making these estimates less reliable and more difficult to generalize.

The leave-one-out analysis reassured us that no individual study had a substantial influence on the pooled prevalence. This suggests that the overall prevalence estimates are robust and not overly influenced by one outlier study, despite the underlying heterogeneity observed between studies.

It should be noted that beyond pooled prevalence estimates in this study, genotypic characterization provides deeper insight into the molecular epidemiology of enteric viruses in the region.

In our analysis, we considered the genetic diversity of cases reported in case-control studies, as these data provide valuable information into the circulation of viral strains. Overall, this review highlights the wide genetic diversity of RVA in Sub-Saharan Africa countries, with thirty-three distinct G/P genotypes reported. This diversity is probably a consequence of co-circulation of multiple viral lineages, high viral load, frequent co-infections, and cross-regional population movements [68]. G1P[8] was the predominant genotype, and reported in eleven countries. This genotype is included in the formulation of the most commonly used RVA vaccines (Rotarix^®^, RotaTeq^®^) [75]. Its predominance suggests that the circulating strains in these countries are generally well matched to the vaccine strains, which may contribute to vaccine effectiveness. Monitoring the distribution of G1P[6] and other genotypes is important to ensure continued protection and to detect any emergence of genotypes not covered by the current vaccines. However, the high frequency of non-vaccine genotypes such as G2P[4] and G12P[8], both present in nine countries, suggests a potential change in genotype dominance following vaccination [68, 76]. It is important to point that G12P[8] is an emerging strain globally recognized, which requires further monitoring [68]. The emergence of unusual genotypes, especially G8P[14], G12P[4] and diverse mixed or non-typable combinations (such as GNTP[6], GxPNT), particularly in Burkina Faso and Kenya, raises concerns about possible interspecies reassortment or underestimated zoonotic transmission [77]. These findings underscore the essential need to strengthen molecular surveillance systems in the region. Moreover, the observed heterogeneity in genotype distribution across countries included in this study reflects variations in genomic surveillance capacity and diagnostic approaches, highlighting the importance of expanding regional genotyping efforts to better monitor epidemiological trends and evaluate vaccine effectiveness against circulating strains [78].

The genotypic distribution of NoV observed in this review confirms the predominance of GII, particularly GII.4, GII.6, and GII.2, which were consistently reported across Burkina Faso, Ethiopia, and South Africa. This is consistent with worldwide trends, which indicate GII.4 as the most prevalent strains around the world due to their high transmissibility and ability to evolve rapidly through antigenic drift and recombination [2, 79]. The detection of several other GII genotypes, including GII.1, GII.3, GII.9, GII.12, GII.14, and GII.16, alongside recombinant strains (such as GII.Pe/GII.4 Sydney 2012, GII.P16/GII.17) reflects the genetic diversity and dynamic evolution of circulating NoV in Africa [4]. Similarly, data from Botswana showed a predominance of GII.4 strains (69.7%), with the recombinant GII.Pe/GII.4 Sydney 2012 varient as the most frequently detected combination, alongside GII.2, GII.12 and GI.9. This finding further supports the regional dominance of GII.4 and ongoing diversification of circulating NoV strains [71]. Research has linked these recombinant variants to periodic global outbreaks, confirming their epidemiological significance [80]. Although detected less frequently, genotype from GI genogroup have been regularly reported in several studies, suggesting a low but persistent circulation of these strains. Reports of mixed infections and identification of recombinant strains, such as GI.f/I.3, have been observed, indicating the possibility of co-infections and inter-genogroup recombination events. Several studies have also documented both intra- and inter-genogroup recombination, highlighting its potential role in antigenic diversification [4]. These mechanisms may further complicate molecular surveillance efforts and represent a challenge for the development of vaccines offering widespread protection.

We observed a moderate genetic diversity of SaV in Sub-Saharan Africa, with data mainly from Burkina Faso, Ethiopia, and Gabon. The detection of multiple genotypes, including from GI and GII, highlights the co-circulation of various SaV lineages in these countries. The predominance of GII.1 in all three countries confirms its widespread distribution in the region and aligns with global trends, where GII.1 and GII.4 genotypes are common [81]. GII.5 and GII.6 have also been detected in several countries, as well as GII.2, GII.3, GII.4, which are less common genotypes, thus further highlighting the diversity of circulating strains [32, 36, 39, 66]. Moreover, the exclusive identification of uncommon genotypes such as GIV.1 and GV.1 in Burkina Faso suggests possible localized emergence or introduction events, which may reflect differences in regional epidemiology [36]. We also identified several GI genotypes in Gabon and Burkina Faso, indicating a heterogeneous viral ecosystem where GI and GII persist, potentially contributing to a larger clinical and evolutionary pattern [36, 66]. Overall, these findings point the importance of ongoing molecular surveillance in Sub-Saharan Africa to better characterize the diversity and distribution of SaV genotypes. Such efforts are essential for understanding viral evolution, transmission dynamics, and the potential impact of genotype variation on disease burden and control strategies.

This study also revealed significant HAstV genetic diversity, particularly in three Sub-Saharan African countries, South Africa, Burkina Faso and Gabon, with HAstV-1 being the predominant genotype. This genotype is known for its well-documented predominance worldwide and association with sporadic gastroenteritis in children [82]. Some genotypes were less common, but although there were in minority, they were reported in epidemic and endemic contexts, suggesting ongoing transmission and the presence of regional variations in strains’ distribution. In this review, Gabon stands out for the presence of non-classical strains belonging to the MLB and VA clades, notably HAstV-VA2 (VA-clade) and MLB1. These emerging HAstV strains have been increasingly reported worldwide and are believed to be responsible for a broader clinical spectrum, including systemic and neurological infections in vulnerable populations [83, 84]. Their identification in Gabon suggests that these emerging lineages may be more widespread in Sub-Saharan Africa. The genetic diversity of HAstVs observed in this review could be explained by different factors such as, improved molecular surveillance, increased diagnostic sensitivity, and the complex interactions between human, environmental, and possibly animal sources. In addition, these findings highlight the need for molecular characterization of circulating strains to monitor the evolution and spread of HAstV genotypes in Africa. And finally, the detection of non-classical strains requires further investigation into their pathogenic potential, transmissibility, and implications for public health interventions, especially in young children.

Our results show a decrease in pooled RVA prevalence form 35% before vaccine introduction to 24% after its introduction. While this crude comparison suggests a significant reduction, the meta-regression analysis, did not find this decline to be statistically significant after accounting for heterogeneity. However impact studies conducted after the official introduction of RVA vaccine into national immunization programs in Sub-Saharan Africa have showed similar results, with a decrease in RVA-associated hospitalizations and mortality following vaccine deployment [68]. Moreover, the substantial heterogeneity observed across the two study periods suggests that factors other than vaccination may have influenced prevalence rates. These could include for example, differences in study design, diagnostic methods, surveillance intensity, or baseline burden, could influence reported prevalence. Thus, countries using only EIA may underestimate the number of cases compared to those incorporating RT-PCR, which may contribute to the observed variability [85]. Moreover, a meta-regression analysis indicated a non-significant mean decrease of 8% points in prevalence after vaccine introduction. Although not statistically significant, this result supports a declining trend in RVA detection post-vaccination. Nevertheless, the low proportion of explained variance indicates that vaccine introduction is not sufficient to explain most observed variations, suggesting the need to take into account contextual factors, such as vaccine coverage rates, and socio-environmental determinants [86]. These findings highlight the need for ongoing surveillance and contextual evaluation of vaccine performance. They also point the importance genomic surveillance, since changes in circulating genotypes could influence vaccine effectiveness and warrant careful monitoring [87, 88].

This study has limitations that should be acknowledged.

First, the review protocol was not prospectively registered in a database such PROSPERO. Although the methodology was defined a priori, the absence of prospective registration may increase the risk of reporting bias and should be considered when interpreting the findings.

Second, publication bias appears to be present, as suggested by the significant results of both Egger’s and Begg’s tests, as well as the visual asymmetry observed in the funnel plot. This may be partly explained by small studies effects or selective reporting. In addition, some studies provided multiple prevalence estimates (for different viruses), which may have exaggerated the apparent asymmetry. Furthermore, despite implementing a comprehensive and structured search strategy, the possibility of incomplete retrieval cannot be fully excluded, as variations in database indexing practices, differences in author terminology, and inherent limitations of search algorithms may have limited the identification of some eligible studies.

Third, although heterogeneity has already been discussed in detail, it remains an important limitation, as it could affect the pooled estimates accuracy and comparability. Third, the geographic distribution of studies was unequal, with a predominance of data from West and Southern Africa, and relatively fewer studies from Central and East Africa. This regional imbalance limits the generalizability of our findings to the broader Sub-Saharan context. Lastly, the small number of studies available for HAstV and SaV makes it difficult to reach definitive conclusions about their prevalence, and limits meaningful comparisons between regions or time frames.

Additionally, most included studies reported prevalence over multi-year periods rather than by individual year, which limited our ability to assess temporal trends in virus prevalence and genotype distribution. Future studies providing annual data would enable more detailed longitudinal analyses and improve understanding of evolving epidemiology.

Based on our findings, we recommend extending epidemiological surveillance and molecular characterization studies to underrepresented regions of Sub-Saharan Africa, especially Central and East Africa. Efforts should also focus on improving the detection and diagnosis of HAstV and SaV, for which data remain limited. In addition, standardization diagnostic methods and study protocols would improve data comparability and support more robust meta-analyses.

Furthermore, integrating genomic approaches such as sequencing and genetic characterization of enteric viruses into surveillance systems, is essential for tracking genotype evolution and detecting new variants. Strengthening the capacity of local molecular biology and bioinformatics laboratories across the continent is also a priority to enable African researchers to independently generate and analyze high-quality data. Finally, integrating enteric virus surveillance into wider public health programs could help improve strategies for prevention, early detection, and control of viral gastroenteritis in the region.

## Conclusion

This systematic review and meta-analysis give a comprehensive overview of the data available on RVA, NoV, HAstV, and SaV in Sub-Saharan Africa over the past decades. It also highlights the genetic diversity of these enteric viruses across the region. Our findings reveal prevalence data for at least one of these viruses in nineteen Sub-Saharan countries, while data on genotypic diversity were available in only thirteen countries. RVA was the most studied virus, followed by NoV, both in terms of prevalence and genotyping. In contrast, very limited information is available on the genetic diversity of HAstV and SaV.

The findings in this review highlight significant knowledge gaps, particularly concerning lesser-studied viruses and regions. These results underscore the need to expand surveillance and molecular characterization efforts to ensure more equitable representation across countries. Such efforts will be critical for informing evidence-based prevention and control strategies adapted to the epidemiological realities of enteric viruses in Sub-Saharan Africa. 

## Supplementary Information

Below is the link to the electronic supplementary material.

Supplementary Material 1

Supplementary Material 2

Supplementary Material 3

Supplementary Material 4

Supplementary Material 5

Supplementary Material 6

Supplementary Material 7

## Data Availability

All data generated or analyzed during this study are included in this published article and its supplementary information files.

## Abbreviations

CI: Confidence interval
Coef.: Coefficient
df: Degrees of freedom
EIA: Enzyme immunoassay
ELISA: Enzyme-linked immunosorbent assay
HAstV: Human astrovirus
ICG: Immunochromatographic test
I2: I-squared statistic (measure of heterogeneity)
JBI: Joanna Briggs Institute
LMICs: Low- and middle-income countries
MLB: Melbourne astrovirus genotype
NoV: Norovirus
NT: Non-typable
PRISMA: Preferred Reporting Items for Systematic Reviews and Meta-Analyses
Q: Cochran’s Q statistic
RVA: Rotavirus A
RT-PCR: Reverse transcription polymerase chain reaction
SaV: Sapovirus
VA: Virginia astrovirus genotype
WASH: Water, sanitation and hygiene
