# Supplementary material for: Prevalence and genetic diversity of enteric viruses in Sub-Saharan Africa: a systematic review and meta-analysis
Source: BMC Infect Dis. 2026 Apr 27;26:1129. doi: 10.1186/s12879-026-13391-7 (PMC13262512; doi:10.1186/s12879-026-13391-7)
Supplement: Supplementary file 1 — Supplementary Material 1 [file 12879_2026_13391_MOESM1_ESM.docx]

Supplementary Table1: Risk of bias assessment of cross-sectional studies using the Joanna Briggs Institute (JBI) Critical Appraisal Checklist

| **Author** | **Q1. Criteria for inclusion clearly defined** | **Q2. Study subjects and setting described in detail** | **Q3. Exposure measured validly and reliably** | **Q4. Objective, standard criteria used for condition measurement** | **Q5. Confounding factors identified** | **Q6. Strategies to deal with confounding factors stated** | **Q7. Outcomes measured validly and reliably** | **Q8. Appropriate statistical analysis used** | **Overall Risk** |
| --- | --- | --- | --- | --- | --- | --- | --- | --- | --- |
| Kurenzvi_2020 | Yes | Yes | Yes | Yes | Unclear | No | Yes | Yes | Moderate |
| Agbla_2020 | Yes | Yes | Yes | Yes | Unclear | Unclear | Yes | Yes | Moderate |
| Nitiema_2011 | Yes | Yes | Yes | Yes | Yes | Unclear | Yes | Yes | Moderate |
| Gelaw_2022 | Yes | Yes | Yes | Yes | Unclear | No | Yes | Yes | Moderate |
| Kaboré_2017 | Yes | Yes | Yes | Yes | Yes | No | Yes | Yes | Moderate |
| Ouédraogo_2016 | Yes | Yes | Yes | Yes | Yes | Yes | Yes | Yes | Low |
| Raini_2015 | Yes | Yes | Yes | Yes | Unclear | Unclear | Yes | Yes | Low |
| Feleke_2018 | Yes | Yes | Yes | Yes | Yes | Yes | Yes | Yes | Low |
| Mwanga_2020 | Yes | Yes | Yes | Yes | Unclear | No | Yes | Yes | Low |
| Lambisia_2020 | Yes | Yes | Yes | Yes | Unclear | No | Yes | Yes | Low |
| Agutu_2017 | Yes | Yes | Yes | Yes | Unclear | No | Yes | Yes | Low |
| Matussek_2015 | Unclear | Yes | Yes | Yes | Yes | Unclear | Yes | Unclear | Moderate |
| Kouéta_2014 | Unclear | Yes | Unclear | Yes | Unclear | Unclear | Unclear | Yes | High |
| Gamsonré_2019 | Yes | Yes | Yes | Yes | Unclear | Unclear | Yes | Yes | Moderate |
| Lompo_2013 | Yes | Yes | Yes | Yes | Unclear | Unclear | Yes | Yes | Moderate |
| Boni-cisse_2018 | Yes | Yes | Yes | Yes | No | No | Yes | Yes | Moderate |
| Binka_2011 | Yes | Yes | Yes | Yes | Unclear | Unclear | Yes | Yes | Low |
| Bawa_2023 | Yes | Yes | Yes | Yes | Yes | Yes | Yes | Yes | Low |
| Enweronu-Laryea_2013 | Yes | No | Yes | Yes | Unclear | Unclear | Yes | No | Moderate |
| Afolabi_2019 | Yes | Unclear | Yes | Yes | Unclear | Unclear | Yes | Yes | Moderate |
| Chigor_2023 | Yes | Yes | Yes | Yes | Unclear | Unclear | Yes | Yes | Moderate |
| Ayolabi_2012 | Yes | Yes | Yes | Yes | Unclear | Unclear | Yes | Yes | Low |
