# Supplementary material for: Prevalence and genetic diversity of enteric viruses in Sub-Saharan Africa: a systematic review and meta-analysis
Source: BMC Infect Dis. 2026 Apr 27;26:1129. doi: 10.1186/s12879-026-13391-7 (PMC13262512; doi:10.1186/s12879-026-13391-7)
Supplement: Supplementary file 2 — Supplementary Material 2 [file 12879_2026_13391_MOESM2_ESM.docx]

Supplementary Table 3: Risk of bias assessment of Prevalence studies using the JBI Critical Appraisal Checklist

| **Study (Author, Year)** | **1. Was the sample frame appropriate to address the target population?** | **2. Were study participants sampled in an appropriate way?** | **3. Was the sample size adequate?** | **4. Were the study subjects and the setting described in detail?** | **5. Was the data analysis conducted with sufficient coverage of the identified sample?** | **6. Were valid methods used for the identification of the condition?** | **7. Was the condition measured in a standard, reliable way for all participants?** | **8. Was there appropriate statistical analysis?** | **9. Was the response rate adequate, and if not, was the low response rate managed appropriately?** | **Overall Risk** |
| --- | --- | --- | --- | --- | --- | --- | --- | --- | --- | --- |
| Maphalala_2018 | Yes | Unclear | Yes | Yes | Yes | Yes | Yes | Yes | Yes | Low |
| Bonkoungou_2011 | Unclear | No | Unclear | Unclear | Unclear | Yes | Yes | Unclear | Unclear | High |
| Bero_2021 | Unclear | Unclear | Unclear | Yes | Unclear | Yes | Unclear | Yes | Unclear | High |
| Nordgren_2012 | Yes | Yes | Yes | Yes | Unclear | Unclear | Yes | Yes | Unclear | Moderate |
| Rönnelid_2020 | Yes | Yes | Yes | Yes | Unclear | Unclear | Yes | Yes | Unclear | Moderate |
| Bonkoungou_2018 | Yes | Yes | Yes | Yes | Unclear | Unclear | Yes | Yes | Unclear | Moderate |
| Potgieter_2023 | Yes | No | Yes | Yes | Unclear | Unclear | Yes | Yes | Unclear | Moderate |
| Khumela_2023 | Yes | Yes | Yes | Yes | Unclear | Unclear | Yes | Yes | Unclear | Moderate |
| Mulondo_2020 | Yes | Yes | Yes | Yes | Unclear | Unclear | Yes | Yes | Unclear | Moderate |
| Mans_2018 | Yes | Yes | Yes | Yes | Yes | Yes | Yes | Yes | Unclear | Low |
| Page_2016 | Yes | Yes | Yes | Yes | Yes | Yes | Yes | Yes | Unclear | Low |
| Ledwaba_2018 | Yes | Yes | Unclear | Yes | Yes | Yes | Yes | Yes | Unclear | Low |
| Mpabalwani et al., 2016 | Yes | Yes | Yes | Yes | Yes | Yes | Yes | Yes | Unclear | Low |
| Mahamadou_2019 | No | Yes | No | Yes | Yes | Yes | Yes | Yes | Unclear | Moderate |
| Mukaratirwa_2014 | Yes | Yes | Yes | Yes | Yes | Yes | Yes | Yes | Unclear | Low |
| Lekana-Douki_2015 | Yes | Unclear | Yes | Yes | Yes | Yes | Yes | Yes | Unclear | Low |
| Mado_2022 | Yes | Yes | Yes | Yes | Yes | Yes | Yes | Yes | Unclear | Low |
| Igwe_2022 | Yes | Yes | Yes | Yes | Yes | Yes | Yes | Yes | Unclear | Low |
| Anochie_2013 | Yes | Yes | Unclear | Yes | Yes | Yes | Yes | Unclear | Unclear | Moderate |
| Aliyu_2017 | Yes | Unclear | Yes | Yes | Yes | Yes | Yes | Yes | Unclear | Low |
| Sanni_2022 | Yes | Yes | Yes | Yes | Yes | Yes | Yes | Yes | Yes | Low |
| Boula_2014 | Yes | Yes | Yes | Yes | Yes | Yes | Yes | Yes | Yes | Low |
| Owor_2018 | Yes | Yes | Yes | Yes | Yes | Yes | Yes | Yes | Yes | Low |
| Hugho_2023 | Yes | Yes | Yes | Yes | Yes | Yes | Yes | Yes | Yes | Low |
| Ogunbiyi_2023 | Yes | Yes | Unclear | Yes | Yes | Yes | Yes | Yes | Unclear | Moderate |
| Tagbo_2019 | Yes | Yes | Yes | Yes | Yes | Yes | Yes | Yes | Unclear | Low |
