# Supplementary material for: Prevalence and genetic diversity of enteric viruses in Sub-Saharan Africa: a systematic review and meta-analysis
Source: BMC Infect Dis. 2026 Apr 27;26:1129. doi: 10.1186/s12879-026-13391-7 (PMC13262512; doi:10.1186/s12879-026-13391-7)
Supplement: Supplementary file 3 — Supplementary Material 3 [file 12879_2026_13391_MOESM3_ESM.docx]

Supplementary table 5: Diversity of NoV genotypes by country

| **NoV genotype** | **Number of countries** | **Countries** | **Reference** |
| --- | --- | --- | --- |
| **GII.4** | 3 | Ethiopia, South Africa, Burkina Faso | [33,35,36,40,52,65] |
| **GII.6** | 3 | Ethiopia, South Africa, Burkina Faso | [33,40] |
| **GI.3** | 3 | Ethiopia, South Africa, Burkina Faso | [35,40,65] |
| **GI.5** | 2 | Ethiopia, Burkina Faso | [33,35,40] |
| **GII.2** | 2 | South Africa, Burkina Faso | [33,65] |
| **GII.3** | 2 | South Africa, Burkina Faso | [35,52] |
| **GII.12** | 1 | Burkina Faso | [35] |
| **GII.1** | 1 | South Africa | [65] |
| **GII.9** | 1 | Burkina Faso | [35] |
| **GII.14** | 1 | Burkina Faso | [35] |
| **GII.16** | 1 | Burkina Faso | [33] |
| **GII.c** | 1 | Burkina Faso | [33] |
| **GII unassigned** | 1 | Burkina Faso | [33] |
| **GI.1** | 1 | Burkina Faso | [33] |
| **GI.f/I.3** | 1 | Burkina Faso | [33] |
| **GI.2** | 1 | Ethiopia | [40] |
