# Supplementary material for: Prevalence and genetic diversity of enteric viruses in Sub-Saharan Africa: a systematic review and meta-analysis
Source: BMC Infect Dis. 2026 Apr 27;26:1129. doi: 10.1186/s12879-026-13391-7 (PMC13262512; doi:10.1186/s12879-026-13391-7)
Supplement: Supplementary file 4 — Supplementary Material 4 [file 12879_2026_13391_MOESM4_ESM.docx]

Supplementary table 4: Summary table of RVA genotypes by country

| **Génotype RVA** | **Number of countries** | **Country** | **Reference** |
| --- | --- | --- | --- |
| G1P[8] | 11 | Benin, Botswana, Burkina Faso, Gabon, Ghana, Kenya, Nigeria, South Africa, Togo, Zimbabwe, Côte d'Ivoire | [11,15,17,18,32–35,39,47,49,51,59,64,66,67] |
| G2P[4] | 9 | Benin, Botswana, Burkina Faso, Gabon, Ghana, Kenya, South Africa, Togo, Zimbabwe | [11,18,34,36,39,47,49,51,59,64] |
| G12P[8] | 9 | Benin, Burkina Faso, Gabon, Ghana, Kenya, Nigeria, South Africa, Togo, Côte d'Ivoire | [11,15,17,30,33,35,36,39,47,59,64,67] |
| G3P[6] | 7 | Benin, Burkina Faso, Gabon, Ghana, Kenya, Nigeria, Côte d'Ivoire | [11,15–18,30,33,43,47,67] |
| G9P[8] | 6 | Burkina Faso, Ethiopia, Kenya, South Africa, Zimbabwe, Ghana | [32–34,39,40,51,59,64] |
| G2P[6] | 6 | Burkina Faso, Ghana, Kenya, South Africa, Zimbabwe, Côte d'Ivoire | [16,18,32,33,51,59] |
| G1P[6] | 5 | Burkina Faso, Ghana, Kenya, Nigeria, Togo | [18,30,35,39,67] |
| G12P[6] | 5 | Burkina Faso, Ghana, Kenya, Togo, Côte d'Ivoire | [15,32,35,36,39,51,66,67] |
| G3P[8] | 5 | Botswana, Burkina Faso, Kenya, South Africa, Ghana | [33,39,49,59,64] |
| G9P[6] | 4 | Burkina Faso, Ghana, Kenya, South Africa | [18,33,39,43,59] |
| G6P[6] | 3 | Burkina Faso, Gabon, Côte d'Ivoire | [33,34,36,47] |
| G2P[8] | 3 | Burkina Faso, Ghana, Kenya | [18,32,33,39,64] |
| G1P[4] | 3 | Burkina Faso, Ghana, Kenya | [18,33,39] |
| G9P[4] | 3 | Burkina Faso, Kenya, South Africa | [33,39,59] |
| G10P[6] | 2 | Burkina Faso, Ghana | [17,18,32] |
| G8P[4] | 2 | Kenya, Tanzania | [39,66] |
| G8P[6] | 2 | Burkina Faso, Kenya | [34,39] |
| G8P[14] | 2 | Kenya | [39] |
| G12P[4] | 1 | Kenya | [39] |
| GNTP[4] | 1 | Kenya | [39] |
| GNTP[6] | 1 | Kenya | [39] |
| GNTP [8] | 1 | Kenya | [39] |
| G1PNT | 1 | Kenya | [39] |
| G8PNT | 1 | Kenya | [39] |
| G9PNT | 1 | Kenya | [39] |
| GNTPNT | 1 | Kenya | [39] |
| G1Pmix | 1 | Ghana | [18] |
| G2Pmix | 1 | Ghana | [18] |
| G4P[9] | 1 | Ghana | [17] |
| G1+G2P[4,8] | 1 | Botswana | [49] |
| G1P[9] | 1 | Burkina Faso | [32] |
| G6P[8] | 1 | Burkina Faso | [36] |
| G8P[8] | 1 | Kenya | [39] |
