# Supplementary material for: Prevalence and genetic diversity of enteric viruses in Sub-Saharan Africa: a systematic review and meta-analysis
Source: BMC Infect Dis. 2026 Apr 27;26:1129. doi: 10.1186/s12879-026-13391-7 (PMC13262512; doi:10.1186/s12879-026-13391-7)
Supplement: Supplementary file 5 — Supplementary Material 5 [file 12879_2026_13391_MOESM5_ESM.docx]

Supplementary table 6: Diversity of SaV genotypes by country

| **SaV genotype** | **Number of countries** | **Countries** | **Reference** |
| --- | --- | --- | --- |
| **GII.1** | 3 | Ethiopia, Burkina Faso, Gabon | [33,37,40,68] |
| **GII.5** | 2 | Ethiopia, Gabon | [40,68] |
| **GII.6** | 2 | Ethiopia, Burkina Faso | [37,40] |
| **GI.2** | 2 | Burkina Faso, Gabon | [33,68] |
| **GI.1** | 1 | Gabon | [68] |
| **GI.3** | 1 | Gabon | [68] |
| **GI.4** | 1 | Burkina Faso | [37] |
| **GII.2** | 1 | Burkina Faso | [33] |
| **GII.3** | 1 | Burkina Faso | [33] |
| **GII.4** | 1 | Burkina Faso | [37] |
| **GIV.1** | 1 | Burkina Faso | [37] |
| **GV.1** | 1 | Burkina Faso | [37] |
