# Supplementary material for: Prevalence and genetic diversity of enteric viruses in Sub-Saharan Africa: a systematic review and meta-analysis
Source: BMC Infect Dis. 2026 Apr 27;26:1129. doi: 10.1186/s12879-026-13391-7 (PMC13262512; doi:10.1186/s12879-026-13391-7)
Supplement: Supplementary file 6 — Supplementary Material 6 [file 12879_2026_13391_MOESM6_ESM.docx]

Supplementary table 7: Diversity of HAstV genotypes by country

| **HAstV Genotype** | **Number of countries** | **Countries** | **Reference** |
| --- | --- | --- | --- |
| **HAstV-1** | 3 | South Africa, Burkina Faso, Gabon | [33,47,56] |
| **HAstV-2** | 3 | Ethiopia, South Africa, Burkina Faso | [33,40,56] |
| **HAstV-5** | 2 | Burkina Faso, Gabon | [33,68] |
| **HAstV-8** | 2 | Burkina Faso, Gabon | [33,68] |
| **HAstV-4** | 1 | Gabon | [47,68] |
| **HAstV-VA2** | 1 | Gabon | [68] |
| **MLB1** | 1 | Gabon | [68] |
