# Supplementary material for: Prevalence and genetic diversity of enteric viruses in Sub-Saharan Africa: a systematic review and meta-analysis
Source: BMC Infect Dis. 2026 Apr 27;26:1129. doi: 10.1186/s12879-026-13391-7 (PMC13262512; doi:10.1186/s12879-026-13391-7)
Supplement: Supplementary file 7 — Supplementary Material 7 [file 12879_2026_13391_MOESM7_ESM.docx]

Supplementary Table 2: Risk of bias assessment of cohort studies using the JBI Critical Appraisal Checklist

| **Study (Author, Year)** | **Q1: Were the two groups similar and recruited from the same population?** | **Q2: Were the exposures measured similarly to assign people to both exposed and unexposed groups?** | **Q3: Was the exposure measured in a valid and reliable way?** | **Q4: Were confounding factors identified?** | **Q5: Were strategies to deal with confounding factors stated?** | **Q6: Were the groups/participants free of the outcome at the start of the study (or at the moment of exposure)?** | **Q7: Were the outcomes measured in a valid and reliable way?** | **Q8: Was the follow up time reported and sufficient for outcomes to occur?** | **Q9: Was follow up complete, and if not, were differences explained?** | **Q10: Were strategies to address incomplete follow up utilized?** | **Q11: Was appropriate statistical analysis used?** | **Overall risk** |
| --- | --- | --- | --- | --- | --- | --- | --- | --- | --- | --- | --- | --- |
| deDeus_2019 | Yes | Yes | Yes | Unclear | Unclear | Yes | Yes | Yes | Unclear | Unclear | Yes | Moderate |
